# Macau, World Capital for Gambling: A Longitudinal Study of a Youth Program Designed to Instill Positive Values

**DOI:** 10.3389/fpubh.2013.00058

**Published:** 2013-11-26

**Authors:** Andrew L. Luk, W. U. Chan, Sydney X. X. Hu

**Affiliations:** ^1^Nethersole Institute of Continuing Holistic Health Education, Hong Kong, China; ^2^Kiang Wu Nursing College of Macau, Macau, China

**Keywords:** adolescents, positive youth development, objective outcome evaluation, subjective outcome evaluation

## Abstract

This study investigated the effectiveness of a positive youth development program for Chinese Secondary 3 students in two schools, who had been followed up since their entry to Secondary 1. A mixed research method was carried out using a pre- and post-test pre-experimental design and a focus group for the participants. The subjective outcome evaluations included participants’ perceptions of the program, program instructors, benefits of the program and overall satisfaction, and were positive. The longitudinal data from the objective outcome evaluation showed some notable improvements, and the overall effect of the program was also found to be positive for newcomers in the junior secondary years. The focus group interviews revealed mostly positive feedback in terms of the students’ general impressions of the program, with the majority of participants perceiving benefits to themselves from the program. The findings offer positive evidence of the effectiveness of the program.

## Introduction

Macau is a small city located near Hong Kong in South East Asia, famous for tourism and its growing gaming industry. In 2011, the estimated population of Macau was 557,400; it has a comparatively young population, those aged between 10 and 24 making up 21% of the total population (1). The Macau Government opened a gaming licensure in 2002, leading to the rapid development of this industry, which has generated a considerable increase of revenue to contribute to the economic growth of Macau, but also may have potentially negative influences on adolescents. Attracted to the employment opportunities and perks in the gaming industry, many adults work in casinos, which require them to work long and irregular hours. One potential implication of this development was highlighted in a government report that clearly identified the problem of lack of communication of parents with their children and the adverse effect on their adolescent development (2). The Youth Indicators published by the Education and Youth Affairs Bureau in 2009 revealed that many teenagers lacked social norms, and that their participation in social functions/affairs and their sense of belongingness to Macau had deteriorated in comparison to an earlier study conducted in 2006 (3). This research also reflects a dramatic increase in youths’ stress levels, originating from pressure at school as well as family conflicts. A recent study has revealed that over half of 744 respondents (54%) agreed that gambling was a common phenomenon in young people in Macau. It is suggested that by building up positive social norms and a sense of morality in adolescents, a more harmonious society in Macau may result (4).

A well-structured local youth program can potentially help adolescents’ positive growth and ensure that they are better prepared for future challenges in life. At present, youth studies and theoretically sound and comprehensive programs for adolescent positive growth and development in Macau are lacking (5, 6). In this study, positive youth development is simply defined as “the growth, cultivation, and nurturance of developmental assets, abilities, and potentials in adolescents” (7). A review by Catalano et al. (8) of 77 programs for positive youth development in North America found that only 25 were successful in terms of positive changes in some objective outcome indicators. However, 15 positive youth development constructs were identified in one or more of the goals of these successful programs. These constructs included: (1) promotion of bonding, (2) cultivation of resilience, (3) promotion of social competence (SC), (4) promotion of emotional competence (EC), (5) promotion of cognitive competence (CC), (6) promotion of behavioral competence (BC), (7) promotion of moral competence (MC), (8) cultivation of self-determination (SD), (9) development of self-efficacy (SE), (10) promotion of spirituality, (11) promotion of beliefs in the future (BF), (12) development of clear and positive identity, (13) recognition for positive behavior, (14) providing opportunities for prosocial involvement (PI), and (15) fostering prosocial norms (PN). With financial support from The Hong Kong Jockey Club Charities Trust through a joint research project consisting of five universities in Hong Kong, a well-tested and comprehensive positive youth development program, “P.A.T.H.S.,” has been developed (9, 10). The word “P.A.T.H.S.” denotes Positive Adolescent Training through Holistic Social Programs, and consists of two tiers of programs. The Tier 1 Program is a universal positive youth development program in which students in Secondary 1–3 participate, normally with 20 h of training in the full program or at least 10 h of training in the core program in each grade. The Tier 1 Program incorporated the 15 positive youth development constructs identified from the existing successful programs (8): Bonding (BO), SC, EC, CC, BC, MC, SE, PN, Resilience (RE), SD, Spirituality (SP), Clear and Positive Identity (ID or CPI), BF, PI, and Recognition for Positive Behavior (PB). All these constructs emphasized helping students to learn and develop their personal autonomy on moral principles, or to make independent and critical judgments via a happy, healthy, and stimulating teaching and learning process during their schooling.

Hong Kong and Macau share a similar Chinese culture, therefore, the well-tested comprehensive positive youth development program “P.A.T.H.S.,” developed for Chinese students in Hong Kong, was modified and adapted for use in Macau. With support from the Education and Youth Affairs Bureau, a local research team was formed by the author and his colleagues, who modified the program so that the content would reflect the local terminology, such as Macau citizens instead of Hong Kong citizens, government structure of Macau Special Administrative Region instead of Hong Kong Special Administrative Region, some indigenous heritage and customs to suit the local context (11). The team also monitored the implementation of the program and evaluated its effectiveness for three consecutive academic years after completion. Two secondary schools were invited to participate as pilot schools to run the program, starting with their Secondary 1 students. Training for teachers and school social workers was also organized both in Hong Kong and Macau. Positive findings of the Secondary 1 and 2 program evaluations during the academic years 2009–2010 and 2010–2011 respectively were reported (11, 12).

Macau has a history of a 15-year free non-tertiary education system, with direct promotion from primary to secondary school without any public examination. Individual admission examination is required after secondary school education for entry into local universities. There is a rather relaxed and less competitive learning atmosphere in Macau’s education system. It is not uncommon to see students repeating their studies in some classes in primary and secondary schools if they find their academic performance unsatisfactory. It is roughly estimated that about 20% of students repeat grades in the junior secondary years in many schools (13). In this study, the effectiveness of the Macau version of the Tier 1 Program of “P.A.T.H.S.” for Secondary 3 students in two pilot schools during the academic year 2011–2012 is evaluated. Since the Secondary 3 classes of the pilot schools included new students who were either repeating the Secondary 3 class or had transferred from other schools, the effectiveness of the program on these new participants was also examined.

## Materials and Methods

A mixed research method was adopted for the triangulation of data. It consisted of a quantitative approach using a pre- and post-tests pre-experimental design, together with a qualitative approach using a participant focus group.

### Participants

The two main sources of data were self-reported questionnaires and focus group discussions. The study participants included all Secondary 1 students in the two chosen schools, School A and School B, totally 232 starting in 2009. These students were followed for 3 years up to and including Secondary 3. When this group of students was promoted to Secondary 2, 53 dropped out of their classes and 79 new students joined the program, either to repeat Secondary 2 or because they were transferred from other schools. When the group was promoted to Secondary 3, 41 dropped out and 48 new students were added (Table 1). Regarding the focus group discussion, two group interviews were conducted, consisting with 8 participants selected randomly from School A in one group and another 8 from School B for the second group.

**Table 1:** **Number of participants and completed questionnaires collected in year 1 (Wave 1 and 2), year 2 (Wave 3 and 4), and year 3 (Wave 5 and 6)**.

	Year 1		Year 2		Year 3	
	Wave 1	Wave 2	Wave 3	Wave 4	Wave 5	Wave 6
Cases of two schools	239	242	268	244	256	240
Successfully matched		232		236		236
Participants joined in Secondary 1			189	173	148	142
Participants joined in Secondary 2			79	63	60	50
Participants joined in Secondary 3					48	44

There were 256 and 240 students who participated in the Wave 5 (W5) pre-test and Wave 6 (W6) post-test respectively. Among the 256 students in the W5 pre-test, 48 (18.75%) were new participants. After discarding the questionnaires that were invalid (mainly due to missing data), 236 questionnaires were successfully matched for analysis. Among these 236 students, 142 had joined the program in Secondary 1, 50 in Secondary 2, and 44 in Secondary 3 (Table 1). There were no significant differences in socio-demographic background between the new (joined in Secondary 3) and old (joined in Secondary 1 and 2) students using the chi-square test, except for age (Table 2). The mean age of the new students (μ ± SD = 16.06 ± 1.17) was higher than that of the old students (μ ± SD = 15.00 ± 1.13).

**Table 2:** **Participant characteristics of P.A.T.H.S. Secondary 3 old and new students**.

Variables	Old (*n* = 208) *n* (%)	New (*n* = 48) *n* (%)	Total (*n* = 256) *n* (%)	*p* Value
Gender				0.109
Male	126 (60.6)	23 (47.9)	149 (58.2)	
Female	82 (39.4)	25 (52.1)	107 (41.8)	
Age				**0.000**
≤13	7 (3.4)	0 (0.0)	7 (2.7)	
14	77 (37.0)	4 (8.3)	81 (31.6)	
15	56 (26.9)	11 (22.9)	67 (26.2)	
16	47 (22.6)	16 (33.3)	63 (24.6)	
17	18 (8.7)	14 (29.2)	32 (12.5)	
≥18	3 (1.4)	3 (6.3)	6 (2.3)	
Family members				0.972
1	1 (0.5)	0 (0.0)	1 (0.4)	
2	10 (4.8)	3 (6.3)	13 (5.1)	
3	48 (23.1)	13 (27.1)	61 (23.8)	
4	97 (46.6)	21 (43.8)	118 (46.1)	
5	40 (19.2)	8 (16.7)	48 (18.8)	
≥6	12 (5.8)	3 (6.3)	15 (5.9)	
Parental marital status				0.544
Divorced	19 (9.4)	5 (11.4)	24 (9.7)	
Separated	12 (5.9)	5 (11.4)	17 (6.9)	
Married	164 (80.8)	33 (75.0)	197 (79.8)	
Family happiness				0.768
Very unpleasant	6 (3.0)	1 (2.3)	7 (2.8)	
Unpleasant	14 (6.9)	5 (11.4)	19 (7.7)	
General	91 (44.8)	17 (38.6)	108 (43.7)	
Pleasant	68 (33.5)	14 (31.8)	82 (33.2)	
Very pleasant	24 (11.8)	7 (15.9)	31 (12.6)	
SSF				0.926
Yes	13 (6.4)	3 (6.8)	16 (6.5)	
No	189 (93.6)	41 (93.2)	230 (93.5)	

*SSF, Social security fund. Significant *p* values are in bold*.

### Instruments

The two set of questionnaires used in Years 1 and 2 were used again when the students were promoted to Year 3. The components of these questionnaires are described below.

### The chinese positive youth development scale

The Chinese Positive Youth Development Scale (CPYDS) is a self-administrated questionnaire developed by Shek et al. (14). It consists of 15 subscales (90 items) that address the 15 constructs of the program: BO (6 items), SC (7 items), EC (6 items), CC (6 items), BC (6 items), MC (6 items), SE (7 items), PN (5 items), RE (6 items), SD (5 items), SP (7 items), Identity or Clear and Positive Identity (ID or CPI, 7 items), BF (7 items), PI (5 items), and Recognition for Positive Behavior (RP or PB, 4 items). The instrument had good reliability (α = 0.91), ranging from 0.63 to 0.86 (7). The Cronbach’s alpha of the present study was 0.93 and ranged from 0.62 to 0.87. A higher score indicates a higher level of positive youth development.

### Life satisfaction scale

Life satisfaction is another important indicator of positive youth development (15). The five-item Life Satisfaction Scale (LIFE) was developed by Diener et al. (16) to assess a person’s global judgment of his/her own quality of life. The Chinese version was translated by Shek (17) with acceptable psychometric properties. The Cronbach’s alpha of the present study is 0.80. A higher LIFE score indicates a higher level of life satisfaction.

### Behavioral intention scale

The five-item scale was used to assess the adolescents’ behavioral intention to engage in problem behavior, including drinking, smoking, taking drugs, having sex, and gambling. The scale was developed by Shek et al. (7) and has good reliability (α = 0.84). The Cronbach’s alpha of the present study is 0.71. A higher Behavioral Intention Scale (BI) score indicates a higher behavioral intention.

### School adjustment measures

The scale was developed by Shek (18) and has good reliability (α = 0.73). The school adjustment measures (SA) consist of three items. Two assess the participant’s perception of his/her academic performance. The third assesses the participant’s perception of his/her conduct. Previous studies have shown these measures to be temporally stable and valid (19, 20). The Cronbach’s alpha of the present study was 0.84. In line with other measures, a higher scale score indicates a higher level of school adjustment in this study.

### Subjective outcomes scale (form A)

The Subjective Outcome Evaluation Form (Form A) was designed by Shek and Siu (21). The Form consists of totally 39 items and 4 open questions, which are divided into 5 parts. The first asks for the participants’ views on the program (10 items). The second examines the participants’ views of those involved in delivering the program, including teachers and/or social workers (10 items). The third section examines the participants’ perceptions of the effectiveness of the program (16 items). Three items ask about the likelihood of their joining a similar program in the future, their overall satisfaction with the program, and whether they would recommend it to others. The final part consists of four open questions on things that participants have learned and appreciated most, as well as their opinions about the instructors and areas for improvement. The Form has good reliability on all 39 items (α = 0.99, mean inter-item correlation = 0.80) (22). The present study has a Cronbach’s alpha of 0.98, with mean inter-item correlation of 0.55.

### Procedures

Quantitative data was collected at two time-points in each year. Firstly, before the program started, the pre-test self-reported questionnaires were completed within 1–2 weeks after the start of the school year. The second data collection time-point occurred at the end of that academic year, after the students had finished the program. The pre-test data Wave 1 (W1) and post-test data Wave 2 (W2) of Secondary 1 were collected and analyzed (11). The pre-test data Wave 3 (W3) and post-test data Wave 4 (W4) of Secondary 2 were analyzed and the results were published (12). These data were used as the baseline for the longitudinal assessment for the Secondary 3 year. The pre-test data W5 and post-test data W6 of Secondary 3 were collected in this study to assess the effectiveness of the Secondary 3 program.

At the pre- and post-tests, the participants were invited to complete a valid and reliable questionnaire, including measures of positive youth development, life satisfaction, school adjustment, adolescent problem behaviors, and demographic information. An identical questionnaire was used in the pre- and post-tests. After completion of the program each year, an evaluation questionnaire was also completed by the participants to assess their satisfaction with the course and perceived benefits of the program, providing subjective outcome measures for evaluation.

The focus group interview took about 1 h each time. The principal investigator conducted the focus group interview using a semi-structured interview guide provided by the Hong Kong research team.

### Data analysis

Descriptive statistics were used to present the subjective outcome measures. The paired-samples *t*-test, one-way ANOVA, and repeated ANOVA were performed to examine differences between the scales, providing objective outcome measures for evaluation. Regarding the qualitative data, the content of the interviews was audio-taped with the consent of the participants. It was then transcribed by the research assistant and checked for accuracy by the principal investigator. The raw data of the two groups were analyzed together through coding. After comparison of all the coding, relevant themes were developed.

## Results

### Subjective outcome evaluation

Table 3 shows the participants’ perception of the program and its instructors. Since the Likert scales used for this section of questionnaire were from 1 to 6, the proportion of responders who endorsed 1, 2, or 3 for disagreement were summed and compared to the proportion of responders who endorsed 4, 5, 6 for agreement. The results showed that most of the students evaluated the program positively. With reference to the views of the course, the most positive response was that there was much peer interaction among students (84.1% agreed responses; *M* = 4.53). The least positive one was overall, I have a very positive evaluation of the program (73.1% agreed responses; *M* = 4.03). With regard to the views of the instructors, the most positive response was they felt that the instructors encouraged them to participate in the activities (92.9% agreed responses; *M* = 4.71). The least positive one was the instructors’ teaching skills were good (79.2% agreed responses; *M* = 4.30).

**Table 3:** **Findings from the subjective outcome evaluation (*n* = 241)**.

		Percentage of responses (%)		*M*	SD
		A	B	
**YOUR VIEWS OF THE COURSE(S)**					
1	The objectives of the curriculum are very clear	18.3	81.7	4.27	1.01
2	The design of the curriculum is very good	22.4	77.7	4.14	1.10
3	The activities were carefully planned	23.6	76.4	4.13	1.11
4	The classroom atmosphere was very pleasant	22.8	77.1	4.21	1.25
5	There was much peer interaction amongst the students	15.8	84.1	4.53	1.13
6	I participated actively during lessons (including discussions, sharing, games, etc.)	22.8	76.7	4.17	1.24
7	I was encouraged to do my best	22.4	77.6	4.15	1.18
8	The learning experience I encountered enhanced my interest in the lessons	25.7	73.5	4.09	1.29
9	Overall, I have a very positive evaluation of the program	26.6	73.1	4.03	1.30
10	On the whole, I like this curriculum very much	23.6	75.9	4.18	1.35
**YOUR VIEWS OF THE INSTRUCTOR(S)**					
1	The instructor(s) had a good mastery of the curriculum	13.7	86.3	4.40	1.10
2	The instructor(s) was/were well prepared for the lessons	12.0	88.0	4.51	1.10
3	The instructor(s)’ teaching skills was/were good	20.7	79.2	4.30	1.20
4	The instructor(s) showed good professional attitudes	9.1	90.8	4.59	1.07
5	The instructor(s) was/were very involved	8.3	91.7	4.65	1.02
6	The instructor(s) encouraged students to participate in the activities	6.6	92.9	4.71	0.95
7	The instructor(s) cared for the students	7.5	92.5	4.71	0.99
8	The instructor(s) was/were ready to offer help to students when needed	7.9	91.7	4.72	0.97
9	The instructor(s) had much interaction with the students	13.6	86.4	4.51	1.16
10	Overall, I have a very positive evaluation of the instructors	9.2	90.8	4.67	1.07

*1, Strongly disagree; 2, disagree; 3, slightly disagree; 4, slightly agree; 5, agree; 6, strongly agree; A, sum of the disagree responses (1 + 2 + 3); B, sum of the agree responses (4 + 5 + 6)*.

As far as the participant’s perception of the benefits of the Tier 1 program was concerned, since the Likert scales used for this section of questionnaire were from 1 to 5, the proportion of responders who endorsed 1 and 2 were summed as unhelpful responses and compared to the proportion of responders who endorsed 4 and 5 for helpful responses. After discarding the middle score of 3, the scales would be symmetrical for comparison. The results showed that all participants evaluated the course as having more helpful than the unhelpful responses. The most helpful response was that it has enhanced their SC (53.9% helpful responses; *M* = 3.48). The least helpful one was that it has strengthened their bonding with teachers, classmates, and their family (39.9% helpful responses; *M* = 3.23) (Table 4). Regarding other aspects of the evaluation, most of the participants indicated that they would recommend the program to their friends who had needs and conditions similar to them (74.2%), and over 60% of them would consider joining similar courses in the future (64.8%) (Table 5). On the whole, a vast majority of them were satisfied with this course (87.1%). The qualitative analysis of the four open-ended questions will not be reported in this paper.

**Table 4:** **Perceptions of the extent to which the course has helped them (*n* = 241)**.

The extent to which the course has helped you		Percentage of responses (%)		*M*	SD
		A	B	
1	It has strengthened my bonding with teachers, classmates, and my family	21.2	39.9	3.23	1.01
2	It has strengthened my resilience in adverse conditions	15.3	45.3	3.42	1.01
3	It has enhanced my social competence	14.1	53.9	3.49	0.96
4	It has improved my ability to handle and express my emotions	16.2	45.6	3.39	0.95
5	It has enhanced my cognitive competence	14.1	48.6	3.48	0.99
6	My ability to resist harmful influences has improved	16.2	46.1	3.41	1.04
7	It has strengthened my ability to distinguish between good and bad	15.3	47.3	3.43	0.98
8	It has increased my competence in making sensible and wise choices	15.8	48.9	3.44	1.03
9	It has helped me to have life reflections	17.1	50.6	3.42	1.08
10	It has reinforced my self-confidence	18.7	44.8	3.35	1.11
11	It has increased my self-awareness	19.9	48.2	3.38	1.10
12	It has helped me to face the future with a positive attitude	16.6	49.4	3.46	1.08
13	It has helped me to cultivate compassion and care for others	17.4	44.8	3.38	1.05
14	It has encouraged me to care about the community	20.8	44.4	3.33	1.06
15	It has promoted my sense of responsibility in serving society	19.9	43.6	3.33	1.03
16	It has enriched my overall development	17.4	48.1	3.47	1.07

*1, Unhelpful; 2, not very helpful; 3, slightly helpful; 4, helpful; 5, very helpful; A, sum of the unhelpful responses (1 + 2); B, sum of the helpful responses (4 + 5)*.

**Table 5:** **Other aspects of subjective outcome evaluation**.

		Percentage of responses (%)		*M*	SD
		A	B	
3	If your friends have needs and conditions similar to yours, will you suggest that they join this course?	23.3	74.2	2.86	0.81
4	Will you participate in similar courses again in the future?	32.8	64.8	2.71	0.81
5	On the whole, are you satisfied with this course?	12.1	87.1	4.29	1.06

*1, Definitely will not; 2, will not; 3, will; 4, definitely will; A, sum of those who will not (1 + 2); B, sum of those who will (3 + 4); 1, very dissatisfied; 2, moderated dissatisfied; 3, slightly dissatisfied; 4, satisfied; 5, moderately satisfied; 6, very satisfied; A, sum of those dissatisfied (1 + 2 + 3); B, sum of those satisfied (4 + 5 + 6)*.

### Objective outcome evaluation

**Figure 1:**
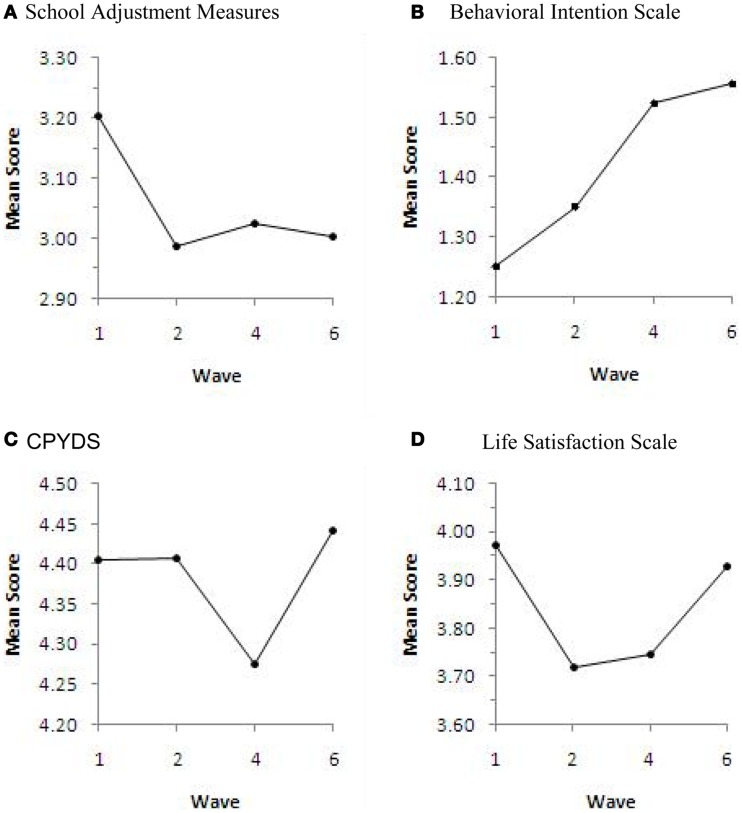
**The participants’ response based on different scales at different time-points**. Wave 1, Secondary 1 pre-test (baseline); Wave 2, Secondary 1 post-test; Wave 4, Secondary 2 post-test; Wave 6, Secondary 3 post-test.

For the objective evaluation of the Secondary 3 program, a paired *t*-test was used. Table 6 highlights the changes between the pre-test (W5) and the post-test (W6) for participants who had joined the program in Secondary 3. There was a significant improvement of the score of the CYPDS, with 8 of the 15 subscales (RE, SC, RP, EC, CC, MC, SD, PN) and SA found to have significant positive changes. For the students who had joined the program before Secondary 3, significant positive changes were also found in the score of CYPDS, three of the subscales (RE, CC, ID), and LIFE (Table 7). However, the SA score was found to have decreased significantly. Table 8 shows the differences between W5 and W6 among all the participants. On the whole, significant improvements were found in CYPDS, eight of the CYPDS subscales (RE, SC, EC, CC, MC, SD, ID, PN) and LIFE. To investigate the outcomes against the length of time participating in the program, the W6 data was analyzed using one-way ANOVA. Non-significant results were revealed in all the scales except BI (*F* = 3.86, *df*  = 2.237, *p* = 0.022) (Table 9). To assess the longitudinal effect of the program from Secondary 1 to 3, a repeated ANOVA was done using W1 (baseline), W2, W4, and W6 data after the participants had completed all 3 years of program. Significant results were found in the CYPDS (*F* = 4.42, *df*  = 3.128, *p* = 0.006), LIFE (*F* = 3.10, *df*  = 3.131, *p* = 0.035), SA (*F* = 5.34, *df*  = 3.126, *p* = 0.001), and BI (*F* = 24.90, *df*  = 3.131, *p* = 0.000) scales (Table 10). The scoring trends of these scales are shown in Figure 1. The scores of CYPDS and LIFE were found to decrease after the Secondary 1 or 2 program, but increased later after completion of the Secondary 3 course. On the other hand, significant negative changes were observed in SA and BI over the duration of the program.

**Table 6:** **Changes in new participants based on the different objective indicators (*n* = 44)**.

	Pre-test		Post-test		*t-*Value	*p* Value
	*M*	SD	*M*	SD	
CPYDS	4.28	0.54	4.48	0.54	−3.44	**0.001**
BO subscale	4.62	0.75	4.79	0.79	−1.84	0.073
RE subscale	4.53	0.68	4.94	0.67	−3.96	**0.000**
SC subscale	4.40	0.84	4.63	0.74	−2.46	**0.018**
RP subscale	4.09	0.90	4.38	0.92	−2.46	**0.018**
EC subscale	3.87	0.85	4.31	0.90	−4.27	**0.000**
CC subscale	4.24	0.74	4.60	0.62	−3.10	**0.003**
BC subscale	4.27	0.68	4.33	0.60	−0.53	0.601
MC subscale	4.21	0.69	4.50	0.71	−2.63	**0.012**
SD subscale	4.58	0.68	4.90	0.56	−3.23	**0.002**
SE subscale	3.66	0.88	3.54	0.77	0.78	0.441
ID subscale	4.05	0.94	4.23	0.78	−1.31	0.196
BF subscale	4.08	0.90	4.21	0.80	−1.36	0.181
PI subscale	4.32	0.68	4.35	0.98	−0.20	0.844
PN subscale	4.45	0.87	4.75	0.81	−2.35	**0.023**
SP subscale	4.85	1.27	5.06	1.07	−1.33	0.190
LIFE	3.72	0.90	3.95	0.95	−1.48	0.147
SA	2.98	0.75	3.23	0.62	−2.33	**0.025**
BI	1.75	0.71	1.68	0.52	0.71	0.484

*CPYDS, mean of the 15 subscales; BO, bonding; SO, social competence; EC, emotional competence; CC, cognitive competence; BC, behavioral competence; MC, moral competence; SE, self-efficacy; PN, prosocial norms; RE, resilience; SD, self-determination; SP, spirituality; ID, identity or clear and positive identity; BF, beliefs in the future; PI, prosocial involvement; RP, recognition for positive behavior; LIFE, life satisfaction scale; SA, school adjustment measures; BI, behavioral intention scale. Significant *p* values are in bold*.

**Table 7:** **Changes in old participants based on the different objective indicators (*n* = 192)**.

	Pre-test		Post-test		*t-*Value	*p* Value
	*M*	SD	*M*	SD	
CPYDS	4.35	0.56	4.42	0.58	−2.41	**0.017**
BO subscale	4.49	0.79	4.52	0.78	−0.59	0.556
RE subscale	4.54	0.73	4.66	0.75	−2.57	**0.011**
SC subscale	4.41	0.71	4.47	0.78	−1.19	0.234
RP subscale	4.14	0.86	4.19	0.85	−0.73	0.465
EC subscale	4.22	0.80	4.32	0.85	−1.69	0.092
CC subscale	4.42	0.75	4.52	0.81	−2.14	**0.034**
BC subscale	4.36	0.72	4.43	0.65	−1.66	0.098
MC subscale	4.38	0.66	4.45	0.70	−1.41	0.159
SD subscale	4.67	0.66	4.71	0.73	−0.80	0.424
SE subscale	3.81	0.86	3.87	0.88	−1.00	0.317
ID subscale	4.09	0.76	4.20	0.77	−2.07	**0.039**
BF subscale	4.26	0.78	4.28	0.81	−0.44	0.661
PI subscale	4.19	0.87	4.28	0.95	−1.40	0.164
PN subscale	4.56	0.77	4.63	0.76	−1.39	0.167
SP subscale	4.86	1.14	4.88	1.11	−0.32	0.749
LIFE	3.70	0.96	3.83	0.94	−1.98	**0.049**
SA	3.08	0.61	2.97	0.64	2.34	**0.020**
BI	1.57	0.51	1.61	0.58	−1.16	0.246

*CPYDS, mean of the 15 subscales; BO, bonding; SO, social competence; EC, emotional competence; CC, cognitive competence; BC, behavioral competence; MC, moral competence; SE, self-efficacy; PN, prosocial norms; RE, resilience; SD, self-determination; SP, spirituality; ID, identity or clear and positive identity; BF, beliefs in the future; PI, prosocial involvement; RP, recognition for positive behavior; LIFE, life satisfaction scale; SA, school adjustment measures; BI, behavioral intention scale. Significant *p* values are in bold*.

**Table 8:** **The changes of all participants based on the different objective indicators (*n* = 236)**.

	Pre-test		Post-test		*t-*Value	*p* Value
	*M*	SD	*M*	SD	
CPYDS	4.34	0.55	4.43	0.57	−3.57	**0.000**
BO subscale	4.52	0.78	4.57	0.78	−1.22	0.222
RE subscale	4.54	0.72	4.71	0.74	−4.01	**0.000**
SC subscale	4.41	0.73	4.50	0.77	−2.08	**0.039**
RP subscale	4.13	0.87	4.22	0.86	−1.71	0.089
EC subscale	4.16	0.82	4.32	0.86	−3.15	**0.002**
CC subscale	4.38	0.75	4.53	0.78	−3.37	**0.001**
BC subscale	4.34	0.71	4.41	0.64	−1.70	0.090
MC subscale	4.35	0.67	4.46	0.70	−2.52	**0.013**
SD subscale	4.65	0.67	4.75	0.70	−2.06	**0.041**
SE subscale	3.78	0.86	3.81	0.87	−0.42	0.673
ID subscale	4.08	0.79	4.20	0.77	−2.46	**0.015**
BF subscale	4.22	0.80	4.27	0.81	−0.92	0.357
PI subscale	4.21	0.84	4.29	0.95	−1.33	0.183
PN subscale	4.54	0.79	4.65	0.77	−2.38	**0.018**
SP subscale	4.86	1.17	4.91	1.10	−0.92	0.361
LIFE	3.71	0.95	3.85	0.94	−2.46	**0.015**
SA	3.06	0.64	3.02	0.64	0.93	0.355
BI	1.60	0.56	1.62	0.57	−0.54	0.588

*CPYDS, mean of the 15 subscales; BO, bonding; SO, social competence; EC, emotional competence; CC, cognitive competence; BC, behavioral competence; MC, moral competence; SE, self-efficacy; PN, prosocial norms; RE, resilience; SD, self-determination; SP, spirituality; ID, identity or clear and positive identity; BF, beliefs in the future; PI, prosocial involvement; RP, recognition for positive behavior; LIFE, life satisfaction scale; SA, school adjustment measures; BI, behavioral intention scale. Significant *p* values are in bold*.

**Table 9:** **The comparison of objective outcomes based on participating time (*n* = 240)**.

	Joined in Secondary 1 (*n* = 143)		Joined in Secondary 2 (*n* = 51)		Joined in Secondary 3 (*n* = 46)		*F-*value	*p* Value
	*M*	SD	*M*	SD	*M*	SD	
CPYDS	4.43	0.59	4.39	0.55	4.45	0.53	0.17	0.842
LIFE	3.90	0.88	3.64	1.07	3.92	0.95	1.66	0.193
SA	2.96	0.65	2.99	0.62	3.20	0.62	2.45	0.088
BI	1.54	0.53	1.79	0.67	1.67	0.51	3.86	**0.022**

*Significant *p* value is in bold*.

**Table 10:** **The continuous effect of P.A.T.H.S. based on different scales**.

	Wave 1		Wave 2		Wave 4		Wave 6		*F*-value	*p* Value
	*M*	SD	*M*	SD	*M*	SD	*M*	SD	
CPYDS (*n* = 131)*	4.40	0.54	4.41	0.58	4.27	0.66	4.44	0.60	4.42	**0.006**
LIFE (*n* = 134)*	3.97	0.99	3.72	1.00	3.75	1.12	3.93	0.88	3.10	**0.035**
SA (*n* = 129)*	3.20	0.63	2.99	0.62	3.02	0.65	3.00	0.60	5.34	**0.001**
BI (*n* = 134)*	1.25	0.38	1.35	0.42	1.52	0.51	1.56	0.54	24.90	**0.000**

*p Value of Mauchly’s test of sphericity <0.05, Greenhouse–Geisser used. Significant *p* values are in bold.

### Findings of the focus group interviews

Eight students from School A, four male and four female, participated in one of the group interviews. Two were very quiet and seldom responded to the interviewer. Eight students from School B, six male and two female, participated in the other group interview. All were quite responsive to the group discussion. The qualitative findings were mainly analyzed in two areas: the participants’ general impression of the program and perceived benefits of the program to themselves. The preliminary analyses were classified into positive and negative comments on the program. Regarding their general impressions, among the 14 who gave feedback, 4 claimed that they felt bored, lacked time in 1 session, and felt tired toward the end of the day when the program was conducted or the content had been repeated with less input; the rest gave positive responses such as that the content met their daily needs, that it was not as boring as other subjects, that it was interesting, relaxing, interactive, free communication, and an enjoyable small group discussion. The activities that aroused their interest were games, videos, success stories, experiences shared by instructors, and incentives provided during the course. With reference to the perceived benefits, eight students who gave positive feedback asserted that through the program, they had learned how to establish goals for the future, to see things from different angles, to make decisions, to control their emotions, and to develop healthy relationships with others. Some of their narratives were as follows:

There was one session about how to develop your future. I might be a bit puzzled about my own future. So, the worksheet in that session helped me to establish personal goals and dreams. – From a student in School A.

Once I was taught to see things from different angles, I practiced what I had been taught and found that things were really different when you saw them from different perspectives. – From a student in School A.

One session talked about romance. If I have to choose a girl or a boy, it is possible that I will remember the principles from that session, and that they will help me to make the right decision. – From a student in School A.

It is helpful to manage one’s emotions. We were taught some techniques, such as eating, listening to music, etc. Listening to music helps to release emotions. It has a calming influence. – From a student in School B.

My temper has improved somewhat. I remember being taught not to be unhappy due to being angry at others. – From a student in School B.

In our age group, dating is common, and we think it is very important. But we do not know how to manage broken relationships. Some even think of committing suicide. Through this program, we learned that even without love from the opposite sex, we still have friendships, and love from our family members. – From a student in School B.

## Discussion

In this study, the participants’ perceptions of the program and of their program instructors were positive. With regard to their perceptions of the effectiveness of the program and their overall satisfaction, the feedback was also positive. On the whole, the subjective outcome evaluations generally supported positive perceptions of the program, program instructors, benefits of the program and overall satisfaction with the whole course, and these findings were consistent with previous results from the programs for Secondary 1 and 2 in Macau (11, 12), and those reported in Hong Kong (22, 23). Though the results in Hong Kong were better than those in Macau, it should be noted that it was reasonable to sum the proportion of responders who endorsed 1, 2, or 3 and compare them to the proportion of responders who endorsed 4, 5, 6 since the scales were symmetrical. On the other hand, it was inappropriate to sum the proportion of respondents that endorsed 1 and 2 and compare to the proportion that endorsed 3, 4, or 5, which might give an exaggerated figure since they were not symmetrical. One of the subscales in the Hong Kong study was calculated in this manner which might present an overly positive picture. Referring to their views of the instructors, fewer participants agreed that the instructors’ teaching skills were good. This might be one of the reasons why some participants claimed that they felt bored when interviewed in the focus groups. There may be several possible reasons for this view. First, participants in Secondary 3 were older; they may have had more expectations of their instructors. Second, since the same 15 constructs are repeated each year but with more in-depth exploration for the senior class, more knowledge and skill are required of the instructors in order to integrate and apply the constructs to the different levels. In future, more training should be provided to instructors, and sharing among peers will help enrich knowledge and experiences to enhance the teaching skills needed to conduct this type of adolescent program. However, when viewing the most positive responses of the two main sets of subjective data, most participants agreed that the program provided much peer interaction amongst them and the instructors encouraged their participations, which were internally consistent with their perception that the program has enhanced their SC. Besides, the objective outcome evaluation and the qualitative data provide alternative evidence to support a positive view.

With regard to the objective outcome evaluation, our findings showed that the Secondary 3 program was effective for newly joined participants, with improvements in more than half of the subscales. This was consistent with the findings of the Secondary 2 program, which was also effective for new participants (12). Possible reasons may be related to its new, interactive, and non-academic nature. In addition, great improvement was also found in their school adjustment. The program may have had some effect on their conduct, while repeating Secondary 3 may have helped to improve their academic performance. With reference to the old participants who had joined the program before Secondary 3, positive changes were also found. However, there was a decrease in the school adjustment score in this group; one possible reason may be the higher academic demands placed on them when they are promoted to the senior class in secondary school. Viewing the old and new participants as a single group, we can see that there was a significant improvement in development and life satisfaction, which is consistent with the results in Hong Kong (24). However, when comparing the Secondary 3 students in Macau with means of CPYDS = 4.44, BI = 1.56 with the Hong Kong group of students with means of CPYDS = 4.54, BI = 1.44 (24), the students in the Hong Kong group showed a slightly better improvement than those in Macau. It would be interesting if the “before score” in Hong Kong could also be compared with the “before score” in Macau to see the real differences and to explore the reasons behind them. This area is therefore worthy of further exploration.

As far as the duration of participating in the program is concerned, joining in either the Secondary 1, 2, or 3 program and using the W6 as the final exit point, no significant differences in the development scales or other measures were found, which shows that participants can join at different times, with a final effective result. The findings revealed both the short- and long-term effects of the 3-year programs on the program participants. In other words, participants can benefit from joining either one, two, or all three programs. On the other hand, there was a significant difference in the problem behavior intention. Participants joining the program for three consecutive years starting from Secondary 1 displayed a lower level of intention to engage in problem behavior than did students joining in Secondary 2 and 3, which is also consistent with the studies in Hong Kong (25) that found that the program can be a protective factor in preventing adolescent problem behavior. It also showed that the program can be more beneficial if students can participate at an earlier age. When assessing the longitudinal effect of the program from Secondary 1 to 3, there was a positive result on the development scale and life satisfaction, which is consistent with the findings in Hong Kong (24). The findings once again revealed the effectiveness of the modified program in Macau with the positive results of the Secondary 1 and 2 programs (11, 12). Although there were negative changes in school adjustment and problem behavior intention, as indicated in the longitudinal study in Hong Kong (25), the program can still be a protective mechanism in delaying adolescent problems.

### The focus group interviews

Regarding the general impression of the program, only a few of the participants perceived it to be boring. Most of them found the program more relaxing and interesting than their conventional moral or civics classes or other formal classes. The class was interactive, with a variety of activities and free communication in small groups. They enjoyed games and video shows that were more up-to-date and related to their daily life. It is consistent with the observation of Shek (24) that only a small portion of participants, approximately 15%, failed to perceive the program as effective. With reference to the perceived benefits of the program, all participants were positive in their feedback. Generally speaking, benefits were observed on both personal and interpersonal levels. The focus group observations were generally consistent with both the subjective and objective outcome evaluation findings in this study, with the students moving in a positive direction in various developmental domains such as establishing personal goals, controlling their emotions, and developing rational thinking and healthy interpersonal relationships. Some suggestions for improving the program were also obtained from the focus groups, such as increasing outdoor activities and extending the time of each session to facilitate more in-depth discussion.

On the whole, the program is effective due to its non-academic, informal, and interactive nature, which is more receptive by the adolescents in building up their positive potentials. On the other hand, more attention should be paid to the behavioral intention of having drugs, sex, and gambling, which may be due to the different messages conveyed by the popularity of internet and may not be easily discerned by adolescents nowadays.

### Limitations

This study had several limitations. First, only two schools were involved and the sample size was relatively small, raising the potential for sample bias and making any generalization of the findings difficult. Secondly, the present study was based on a one-group pre-/post-test design, which may not be the most appropriate. Other approaches such as the randomized control trial, which can provide a more rigorous design to give more insight into the effectiveness of an intervention program, could have been considered. Thirdly, a comparatively large portion of students dropped out of the program, which may have affected the longitudinal observation and the observation of long-term effects. It would be worthwhile to follow up this portion of students to see if there is any change in scores afterward. Nevertheless, the present study can contribute to an understanding of the potential benefits of evidence-based youth work, and is also a ground-breaking scientific study showing the impact of the P.A.T.H.S. program on the holistic development of Chinese adolescents in Macau.

## Conclusion

Subjective outcome evaluation highlighted positive results from all participants. The effect of the program from the objective outcome evaluation was found to be positive to both the newcomers and the old students in the junior secondary years. There was also an overall positive feedback from the focus group interviews. Based on the findings from the subjective and objective outcome evaluations and the focus group interviews, it can be concluded that there is positive evidence of the effectiveness of the Secondary 3 program. In addition, the longitudinal data from the objective outcome evaluation did support an improvement from different time spans in the program. It demonstrated that the 3-year P.A.T.H.S. program has both short- and long-term benefits for program participants. However, students who participated in the program for three consecutive years did better than those who took part in only a 1- or 2-year program in terms of the intention to engage in problem behavior. It showed that the Tier 1 program of the P.A.T.H.S. project can help to prevent adolescent problem behavior through promoting positive development. This program is particularly beneficial to the adolescents in Macau since they are more easily to be tempted for gambling as Macau is a world capital for gambling.

### Implications for school health

There are several implications of this study for school health. First, it demonstrates that participating in a youth development program can have both short- and long-term positive effects for personal growth, and that the program can also be a protective factor in preventing adolescent problem behavior. Second, the interactive and non-academic nature of the program can be more easily accepted by participants, especially for Chinese adolescents in Asian countries where teachers are dominant and directive in the Eastern pedagogical culture and academic performance is greatly emphasized (26). Third, since not every youth development program incorporates a mechanism for evaluation, this study shows the importance of evaluating youth programs and provides evidence for the promotion of health during early adolescence.

## Conflict of Interest Statement

The authors declare that the research was conducted in the absence of any commercial or financial relationships that could be construed as a potential conflict of interest.
